# An Inverse Relative Age Effect in Male Alpine Skiers at the Absolute Top Level

**DOI:** 10.3389/fpsyg.2017.01210

**Published:** 2017-07-17

**Authors:** Øyvind Bjerke, Arve Vorland Pedersen, Tore K. Aune, Håvard Lorås

**Affiliations:** ^1^Department of Teacher Education, Norwegian University of Science and Technology Trondheim, Norway; ^2^Department of Neuromedicine and Movement Science, Norwegian University of Science and Technology Trondheim, Norway; ^3^Department of Physical Education and Sport Science, Faculty of Teacher Education and Arts, Nord University Levanger, Norway

**Keywords:** individual sport, performance, elite level, alpine ski racing, talent

## Abstract

The Relative Age Effect (RAE) can be described as the advantage of being born early after a certain cut-off date within a group of selection. The effect has been found across a wide range of sports and is particularly evident in pre-elite sports and team sports with a high selection pressure. At the absolute top level in team elite sports, the advantage of being relatively older has been reported to disappear, and even reverse, so that the relatively younger athletes are advantaged. In order to further examine such a reversal of the RAE, we investigated the performance of the overall top 50 skiers each year in the alpine World Cup, over a period of 20 years, among men (*N* = 234) and women (*N* = 235). The data indicated that the relatively younger male athletes at the absolute top level had accumulated, on average, more World Cup points compared to the relatively older skiers. No such effect was observed among the female skiers. This finding suggest the existence of a reversed relative age effect in male elite alpine skiing.

## Introduction

The relative age effect (RAE) refers to the advantage of being born early after a cut-off date in an annual group. Such grouping is a usual way to organize sport activities (Musch and Grondin, 2001). The RAE increases the likelihood of both performance and selection advantage (Schorer et al., 2013). Especially in sports with a high degree of competition and selection pressure, an overrepresentation of the relatively older athletes within a cohort is usual (Cobley et al., 2009). One explanation for the RAE, is that the relatively older athletes are born nearly 1 year before the youngest in a cohort, being more mature, stronger, and faster than their counterparts (Musch and Grondin, 2001). Consequently, these athletes receive more attention, better training facilities and more training time compared with their peers (Helsen et al., 2005). This explanation is supported by findings of junior elite athletes who are higher, heavier, and stronger than their peers (Musch and Grondin, 2001; Sherar et al., 2007).

The RAE is evident within team sports, like ice hockey, football, and handball (Cobley et al., 2009; Schorer et al., 2009b). Within individual sports, the effects are less consistent, but it is argued that examinations of the RAE in individual sports may uncover the mechanisms more precisely, because the variables that may confound the effects are easier to identify (Baker et al., 2014). Furthermore, the RAE has been shown to be larger within male sport compared to female sport, probably due to stronger competition during developmental stages (Schorer et al., 2009b).

Once established, the RAE is upheld and strengthened by several other factors, such as the fact that selected athletes get access to better training facilities, better coaches, better equipment, etc. (Helsen et al., 1998), known as the Matthew effect (Merton, 1968), and the Pygmalion effect, by which expectations produce changes in achievement (Rosenthal and Jacobson, 1968). Together, with the initial maturational advantage, selection processes affect an individual’s possibilities to invest in sport. Thus, the RAE carries over into adulthood, and has been demonstrated in several studies within a number of sports (Schorer et al., 2009b; Till et al., 2010; Fleming and Fleming, 2012; Steingröver et al., 2017). The RAE found in adults, does naturally not reflect maturational differences in the same way as in adolescent years, since athletes are no longer annually grouped. Rather, it reflects the fact that selection processes during adolescent years, along with the mentioned additional effects, leaves fewer athletes within each cohort who are born late in the year.

While the RAE is often large at the younger levels of sports, the effect is smaller among adults, and may even disappear completely at the elite level (Cobley et al., 2009; Ford and Williams, 2011). Some researchers have even reported an inverse RAE, also within sports with high degree of competition and selection pressure (Schorer et al., 2009b). Ford and Williams (2011) found that the most award-winning athletes and the most valuable players in team sport (e.g., soccer, ice hockey, and baseball players) were more likely to be born late in the selection year. Gibbs et al. (2012) found that the RAE reversed at the most elite level in ice hockey, and that relatively younger players endured a nearly 1 year longer career than their older peers did. Gibbs et al. (2012) reported even that being born at the start of the year reduces the chances of elite play in NHL by Canadian-born players, as they found relatively lower percentages of players born in the 1 months among those selected for NHL All-star rosters, or Olympic rosters. This effect has been referred to as the ‘Underdog-effect’ (McCarthy and Collins, 2014). Another study concluded that relatively younger players in the German soccer Bundesliga earned significantly higher wages compared with relatively older players (Ashworth and Heyndels, 2007), and there are examples that the draftees in ice hockey are relatively younger than their non-drafted peers (Baker and Logan, 2007). One possible explanation for this inverse RAE is that the relatively younger athletes develop superior skills that help them to persist in an unfavorable system.

In alpine skiing, the RAE is also present across all ages and levels. The effect ranges from the youngest national level (Müller et al., 2015), via the youth level in Winter Olympic Games (Raschner et al., 2012) up to the World Cup level (Müller et al., 2012), and even the top World Cup level (Bjerke et al., 2016). The RAE in alpine skiing is well documented in a recent review (Müller et al., 2016b). However, the RAE seems to diminish within the technical disciplines for World Cup skiers, while being more prominent within speed disciplines for men, with no such effect among females (Bjerke et al., 2016). Thus, like other similar studies, the RAE seems to be smaller at the elite level in alpine skiing than among younger skiers. Previous studies of the RAE at the top level, have included skiers who have had earned at least one World Cup point (Müller et al., 2012) or skiers among the overall top 50 ranked skiers each year (Bjerke et al., 2016). Since the RAE has been shown to diminish at the top level in alpine skiing, and may not even exist within the technical disciplines, the aim of the present study was to examine whether we could find a reversed RAE at the absolute top level in alpine skiing. The present study hypothesized that the RAE might reverse among the very best performances in alpine skiing, defined as the athletes collecting the most World Cup points. Thus, we would see an effect similar to previous studies at the top level (Schorer et al., 2009b; Ford and Williams, 2011). To that end, the data from Bjerke et al. (2016) were re-analyzed in order to tease out a possible inverse RAE.

## Materials and Methods

### Participants

The sample consisted of the top 50 male and top 50 female skiers from each year in the total World Cup ranking list from the period 1995 to 2014, comprising 234 male and 235 female alpine skiers, respectively. The skiers originated from 19 and 21 different nationalities for men and women, respectively, Data were collected from the Fédération Internationale de Ski (FIS) website (FIS, 2014).

### Variables

The skiers’ birthdates were extracted and categorized according to the cut-off date January 1st, which is the international cut-off date for youth skiing (FIS, 2015). Skiers born between January and March comprised Quartile 1, Quartile 2 ranged from April to June, Quartile 3 from July to September, and Quartile 4 from October to December. In addition to date of birth, each individual skier’s performance in the overall World Cup each season, operationalized as World Cup points, where extracted from each of the 20 seasons. This latter measure comprises points collected from all individual disciplines (slalom, giant slalom, Super-G, downhill, and combined) throughout a complete season. As skiers can accumulate World Cup points from several seasons, a summarized World Cup points measure was computed for each individual skier (WCPsum).

### Analysis

The summated World Cup points demonstrated considerable positive skewedness and non-normal distribution according to a significant Kolmogorov–Smirnov test across both male and female sub-samples. Thus, statistical analyses proceeded with non-parametric approaches. In order to investigate whether there was a statistically significant trend between quartiles and overall World Cup points (WCPsum), Jonckheere–Terpstra tests for ordered alternatives were applied with Cohen’s d as measure of effect size for further pairwise comparisons. The statistical analyses were performed in SPSS (Version 21.0, IBM, United States) and *p* < 0.05 was used as statistical significance criterion.

## Results

### Male Elite Alpine Skiers

**FIGURE 1:**
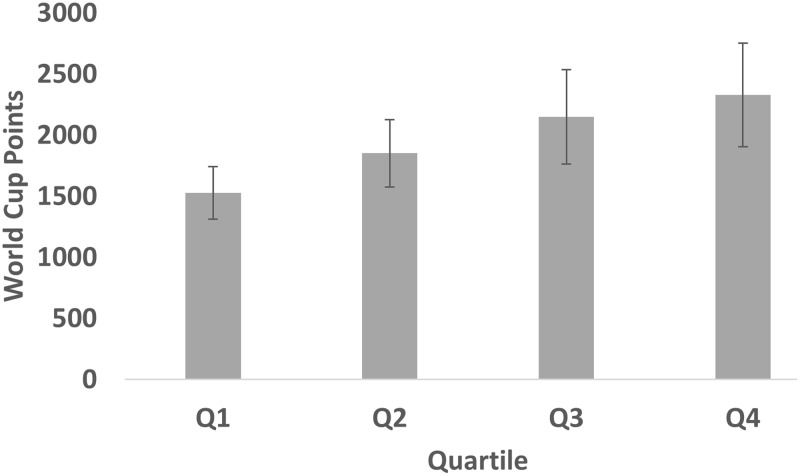
Mean (SEM) World Cup points across quartiles for male elite alpine skiers.

In the cohort of male elite alpine skiers, the average number of World Cup points was 1871 (*SE* = 154) with *SD* = 2359 and median = 998. The distribution of collected WC points, across quartiles, is depicted in **Figure 1**. As is evident, the distribution was not similar with, on average, more points accumulated by skiers born in later quartiles. Consequently, a Jonckheere–Terpstra test for ordered alternatives showed that there was a statistically significant trend of higher median WCPsum scores with later month of birth (from first to fourth quartile) in male skiers, TJT = 569.96, *z* = 2.01, *p* < 0.05, *d* = 0.27. In pairwise comparisons, effect sizes amounted to Q1 vs. Q2: *d* = 0.16; Q1 vs. Q3: *d* = 0.26; Q1 vs. Q4: *d* = 0.32; Q2 vs. Q3: *d* = 0.13; Q2 vs. Q4: *d* = 0.20; Q3 vs. Q4: *d* = 0.06.

### Female Elite Alpine Skiers

**FIGURE 2:**
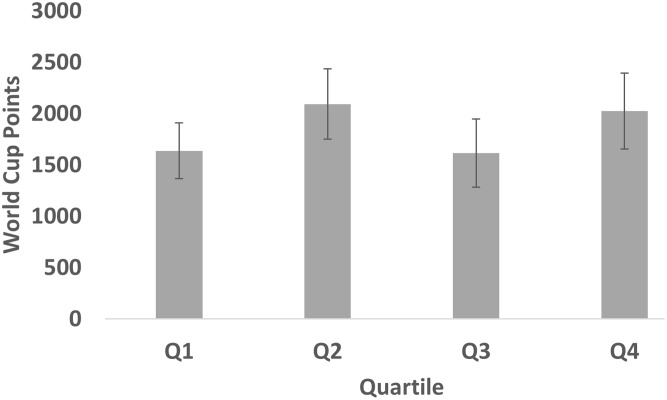
Mean (SEM) World Cup points across quartiles for female elite alpine skiers.

For female elite alpine skiers, the World Cup points amounted to a mean of 1807 (*SE* = 159), *SD* = 2474, and median = 825. The distribution of World Cup points across quartiles for female elite alpine skiers can be found in **Figure 2**. Here, the distribution of points appears to be more similar across quartiles. Indeed, the Jonckheere–Terpstra test for ordered alternatives showed that there was no statistically significant trend of higher median WCPsum scores with later month of birth (from first to fourth quartile) in female skiers, TJT = 582.34, *z* = 1.04, *p* > 0.05, *d* = 0.14. In pairwise comparisons, effect sizes amounted to Q1 vs. Q2: *d* = 0.18; Q1 vs. Q3: *d* = 0.01; Q1 vs. Q4: *d* = 0.16; Q2 vs. Q3: *d* = 0.18; Q2 vs. Q4: *d* = 0.03; Q3 vs. Q4: *d* = 0.16.

## Discussion

The results of the present study demonstrate that there is an inverse RAE at the very top level among male alpine ski racers, previously referred to as the ‘Underdog-effect’ (see Gibbs et al., 2012). Male skiers born late in the year collect, on average, more World Cup points than their earlier born peers. No such inverse effect was found among female skiers. The present data include all skiers placed among the top 50 in the overall World Cup (WC) in any season between 1995 and 2014, and consequently it can be claimed that the data are not only representative for the top level within the sport, but that N, in fact, equals everybody within this particular group of skiers (see Gibbs et al., 2015). As far as we know, this seems to be the first time such a reversal of the RAE has been shown within an individual sport. The same dataset has previously shown a RAE among the male skiers, which was due to the speed discipline specialists (Bjerke et al., 2016). Traditionally, studies of RAE have merely counted the number of subjects belonging to the highest level of performance born in the respective quartiles of the year, while the present study counted the amount of World Cup points collected by skiers born in each quartile. The analysis reversed the RAE such that more points (on average) were collected by skiers in the later quartiles compared with the earlier quartiles.

There is probably several possible reasons why the inverse RAE within alpine skiing at the highest level has not been discovered earlier. Firstly, previous studies have not included skiers at the absolute highest level, and secondly, the measures have not been sufficient for teasing out rather subtle differences. Müller et al. (2012) examined skiers below the absolute top level by including every skier that had taken at least one World Cup point over five seasons from 2006 through 2011. These data included, thus, 742 male and 621 female World Cup skiers. Bjerke et al. (2016) included only skiers who had been among the top 50 in the overall World Cup at least 1 season out of 20, between 1995 through 2014, which reduced the sample to 234 male and 235 female skiers. Each of those skiers had accumulated a minimum of 127 (male) and 117 (female) WC points within a single season, thus representing the absolute top level. As usual in RAE-studies, Bjerke et al. (2016) computed the number of skiers born in each of the respective quartiles, and analyzed differences across quartiles. While Müller et al. (2012) reported a RAE for male WC skiers, Bjerke et al. (2016) reported an overall relative effect for male WC skiers, although the effect disappeared for those skiers specializing in technical disciplines when data were analyzed by skiers’ specialties (speed or technique). We reiterate that the present dataset included the same skiers as Bjerke et al. (2016), but the data were re-analyzed in order to measure the average amount of points collected by skiers born in each respective quartile. Thus, it was possible to detect differences across quartiles for the skiers belonging to the same general (absolute top) level. This procedure is similar to that of Ashworth and Heyndels (2007), who reported an inverse RAE in top level soccer based on the yearly earnings of Bundesliga soccer players, instead of merely counting the number of Bundesliga players born in each quartile, and the study of Ford and Williams (2011), whose data included only award-winning athletes within some of the top competitions.

**FIGURE 3:**
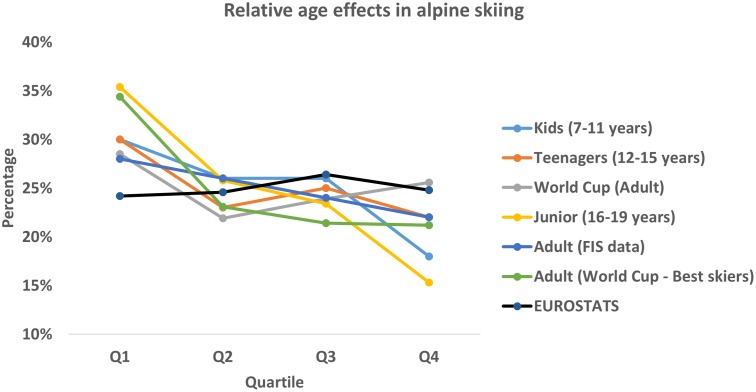
Relative age effects in alpine skiing across ages and samples: Kids/Teenagers from Müller et al. (2015); Adult (World Cup)/Junior from Müller et al. (2012); adult (FIS data) from Baker et al. (2014); adult (World Cup – best skiers) from Bjerke et al. (2016); EUROSTATS – distributions of 53 million live births in the European Union the past decade.

As was shown for handball, by Schorer et al. (2009b), the RAE varies with a number of factors. The RAE was stronger in younger age groups, stronger for male athletes, and weaker or even absent for females. At the absolute top level, the effect was almost non-existent indicating, according to the authors, that non-selected players who stayed in the game had a relatively larger chance of ending up at this level. Similar results were presented for ice hockey by Nolan and Howell (2010), although somewhat rebutted by Gibbs et al. (2012). In alpine skiing, the RAE has been shown to be stronger in younger skiers (Müller et al., 2012, 2016b), and weaker for adult skiers, see **Figure 3**, as well as non-existent for female skiers (Müller et al., 2012; Bjerke et al., 2016). At an even higher level, the effect was non-existent also for male WC skiers specializing into technical disciplines (Bjerke et al., 2016). The present results go even further, and demonstrate an inverse effect for WC skiers at the absolute top level, although not for females.

There are several possible explanations for a reversal of the RAE. Firstly, it has been hypothesized that the effect wanes when the initial physical advantage disappears, and that those who manage to keep up despite their disadvantages do so because they have developed different skills from the relatively older. (Schorer et al., 2009b, 2013). Schorer et al. (2009a) examined, in handball players, whether technical skills could be such a factor, and whether late born players had better technical skills than their earlier born peers, but could not find support for their hypothesis. Their measure of technique, however, included only speed and accuracy of throws so it would seem a bit too early to conclude on technical skills as such. Other studies have focused on variables like perceived competence (Musch and Grondin, 2001), or the age at which athletes begun practicing the sport (Côté et al., 2012) as contenders for explaining why relatively younger athletes overcome the RAE, but no consensus has been reached so far.

Whatever the reason is, it seems that many of the absolute top athletes have come through the ranks despite disadvantages, or adverse incidents, previously in their careers (McCarthy and Collins, 2014; Collins et al., 2016a). It is not, however, possible to conclude from the present data whether the late born skiers have experienced what Collins et al. termed a ‘rocky road,’ and certainly not whether such a ‘rocky road’ is in any way beneficial to athletes, such as sometimes speculated (Schorer et al., 2009b; McCarthy and Collins, 2014; Collins et al., 2016b).

The present inverse RAE is further illustrated, although not statistically backed, by the fact that a large number of the absolute top skiers of all time were, in fact, born in the last (fourth) quartile, contrary to the assumption based on the original RAE. The ski-database ranking system (Alpine Ski Database, 2017) publishes “the super ranking,” a list of the greatest alpine skiers of all time. This list is based upon points calculated together from Olympic Games, World Championships, and World Cup (overall titles, discipline titles, and individual top 10 results). The present top 10 includes seven skiers from the period in question (the last 20 years), who were also included in the present dataset, of whom four (Maier, Svindal, Tomba, Miller) were born in the fourth quartile and have thus defied the RAE. Only one of the seven (Hirscher) was born in one of the first 6 months of the year. Other recent World Cup overall winners, born in the fourth quartile include Kostelic (won the WC in 2011; born in November), and Janka (won the WC in 2010; born in October).

What, then, are the consequences of the RAE, and what might be the consequences of a reversal at the top level? One important consequence of the RAE, is that athletes born early in a cohort are selected based on physical maturity, and that the relatively younger athletes only have a chance of selection if they are early maturing athletes (Gorski et al., 2016; Müller et al., 2016a). Athletes born in the later quartiles are less likely to be selected, and will not have the same access to training facilities and to skilled coaches (Helsen et al., 1998) or to participation in various competitions (Schorer et al., 2009b). This, in turn, may lead to a loss of “talents” in the later adult elite sports because of dropout in the teenage years. The present study, however, may be a positive contribution to the RAE discussion, because the best skiers in the world are on average born late in the year. Instead of a demotivating effect of being born late, this study shows that the persisting skiers become on average better performers than those with the initial advantage of being born early. However, the underlying mechanisms why a reversal occurs are still unclear, and more studies focusing on the mediators of the effect are needed.

## Author Contributions

Conception and design of study: ØB, AVP, TKA and HL; acquisition of data: ØB; analysis and/or interpretation of data: ØB, AVP, TKA and HL; drafting the manuscript: ØB, AVP, TKA and HL; revising the manuscript critically for important intellectual content: ØB, AVP, TKA and HL; approval of the version of the manuscript to be published: ØB, AVP, TKA and HL.

## Conflict of Interest Statement

The authors declare that the research was conducted in the absence of any commercial or financial relationships that could be construed as a potential conflict of interest.
